# Go with the flow: ABCC4 mediates cytokinin efflux to control root development

**DOI:** 10.1093/plphys/kiaf010

**Published:** 2025-01-10

**Authors:** Héctor H Torres-Martínez

**Affiliations:** Assistant Features Editor, Plant Physiology, American Society of Plant Biologists; Department of Biology, Stanford University, Stanford, CA 94305, USA

The plant hormone cytokinin controls many developmental processes in plants, including shoot apical meristem establishment, flower development, leaf cell proliferation, root vascular development, lateral root initiation, and root meristem size (Wybouw and De Rybel 2019). Specifically in root tips, cytokinin plays an important role in establishing meristem size by defining the proximal end of the meristem. For instance, cytokinin signaling induces the onset of the endocycle, a cell cycle where DNA replication is repeated several times without cytokinesis that is associated with cell differentiation and establishing the transition domain (Takahashi et al. 2013). On the other hand, cytokinin acts as a negative regulator of polar auxin transport and promotes auxin degradation in that specific zone (Ioio et al. 2008; Di Mambro et al. 2017). Cytokinin also controls the expression of EXPANSIN A1, facilitating cell wall remodeling in cells that will start to elongate (Pacifici et al. 2018).

The uneven distribution of cytokinins, such as trans-Zeatin (tZ), N^6^-(Δ^2^-isopentenyl)-adenine (iP) and cis-Zeatin, in planta is partly explained by the differential expression of cytokinin synthesis genes (Sakakibara 2021). However, cytokinin transporters also play an important role in mediating the spatial distribution and functional effects of cytokinins on plant development. Significant progress has been made in the past 2 decades in identifying transporters responsible for cytokinin flow. To date, 4 major classes of cytokinin transporters have been identified: PURIN PERMEASE (PUP), EQUILIBRATIVE NUCLEOSIDE TRANSPORTER (ENT), AZA-GUANIN RESISTANT (AZG), and ATP-BINDING CASSETTE (ABC) transporters (Zhang et al. 2023). While most of the identified cytokinin transporters function as importers, little is known about cytokinin efflux carriers, including their location and roles in developmental programs.

In this issue of *Plant Physiology*, Uragami et al. (2024) report on the isolation and characterization of ABCC4, a cytokinin efflux transporter in *Arabidopsis thaliana*, that modulates cytokinin distribution important for root apical meristem size in the primary root. Specifically, ABCC4 appears to be important for defining the transition domain by exporting cytokinin to the apoplast at the proximal end of the root apical meristem, thereby triggering the developmental shift from meristematic cell to cell elongation/differentiation (Fig. 1).

**Figure 1.**
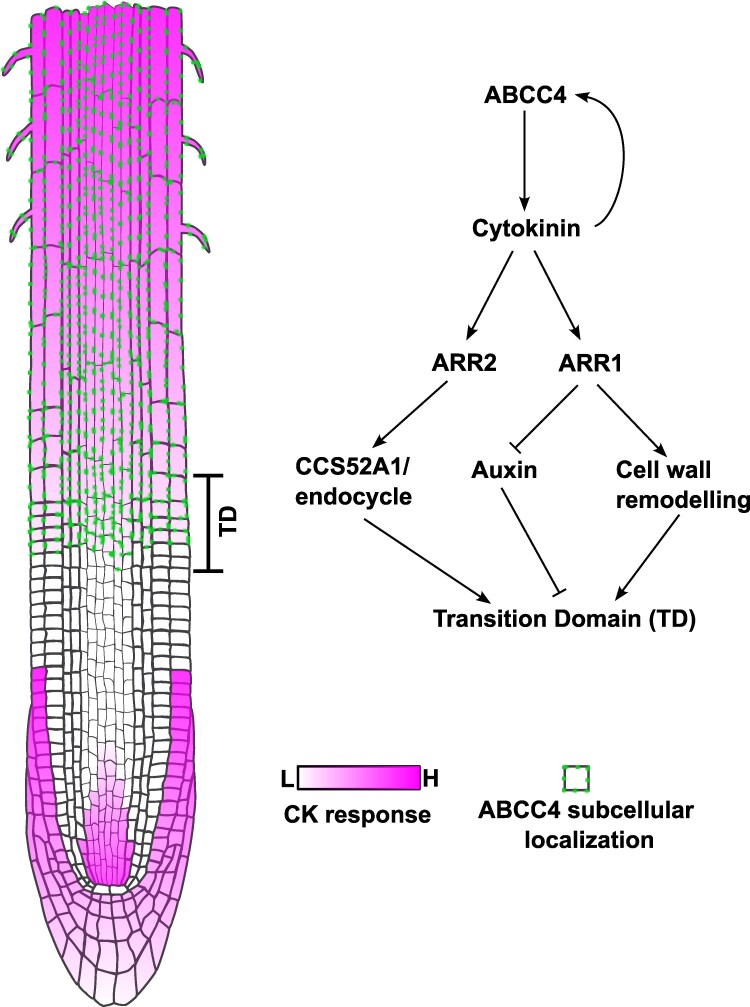
Proposed action mechanism of ABCC4 to regulate root development. In this research, Uragami et al. (2024) uncovered and characterized a new cytokinin efflux transporter that plays an important role in controlling root development. Evidence provided in this work suggests a role for ABCC4 in establishing the transition domain linked to other cytokinin-dependent previously reported mechanisms. Thus, ABCC4 exports cytokinin to the apoplast in the transition domain, and this signal is read out by neighboring cells, which in turn triggers the change of developmental status from a meristematic cell to cell elongation and differentiation. The schematic drawing was made on Inkscape. Cell outlined root tip was created by Frédéric Bouché and is distributed through Bioicons.com under the license CC-BY 4.0 https://creativecommons.org/licenses/by/4.0/.

By heterologous expression analysis coupled with liquid chromatography-mass spectrometry, the authors screened 61 candidate genes derived from a transcriptomic profile study of the shoot apical meristem (Yadav et al. 2009). To this end, the candidate genes were constitutively expressed in tobacco leaves, and aliquots from the incubation buffer were sampled to quantify cytokinins. Tobacco leaves overexpressing *ABCC4* (AT2G47800) showed a significant accumulation of all types of cytokinins compared with the control condition. Time-course experiments showed a progressive accumulation of cytokinins in the incubation buffer. Inhibition experiments with known ABC transporter inhibitors limited the ABCC4-dependant cytokinin accumulation in the incubation buffer.

Arabidopsis transgenic lines with β-estradiol–inducible *ABCC4* expression in MS liquid medium showed a significant increase of iP and cis-Zeatin in the medium after 24 h of treatment with β-estradiol. Similarly, in a heterologous expression system, yeast carrying *ABCC4* under the control of a galactose-inducible promoter showed a significant intracellular reduction of isotope-labeled tZ compared with the control. Together, these results confirm that ABCC4 functions as a cytokinin efflux transporter.

Expression analysis by quantitative reverse transcription polymerase chain reaction (qRT-PCR) showed that *ABCC4* is predominantly expressed in roots during early developmental stages but broadly expressed across the plant at later stages. These results confirmed what was previously reported in a transgenic line where GUS activity was driven by *ABCC4* promoter (Klein et al. 2004). Roots treated with tZ and iP for 2 h showed striking induction of *ABCC4* expression, while auxin, Indole-3-acetic acid (IAA) treatment suppressed *ABCC4* expression. This confirms that cytokinins explicitly induce transporter expression.

T-DNA insertion and CRISPR/Cas9 loss-of-function mutants *abcc4-1* and *abcc4-2*, respectively, exhibited significantly longer primary roots with an increased root apical meristem size. When mutants were grown on tZ-supplemented media, the elongated root phenotype was abolished, indicating that cytokinin transport deficiency is responsible for *abcc4* mutants’ phenotype. Conversely, *ABCC4* overexpressing lines showed reduced primary root and root apical meristem sizes. Interestingly, the lateral root growth rate was higher in overexpressing lines.

Finally, analysis of the activity of a synthetic cytokinin response promoter, *TCSn* (Zürcher et al. 2013), in *abcc4-1* and overexpressing line backgrounds showed altered distribution of *TCSn* activity in the root tip epidermis relative to wild-type plants, suggesting that *ABCC4* regulates cytokinin distribution at a cellular level in root tips. To investigate whether the ABCC4 transporter contributes to root-to-shoot cytokinin transport, the authors applied tZ to roots of *abcc4-1*, overexpressing line, and wild-type plants. They then looked at expression levels of *ARABIDOPSIS RESPONSE REGULATOR5* (*ARR5*), a member of a cytokinin-responsive transcription factor family (D’Agostino et al. 2000) in the shoots. In all 3 genotypes, there was an increase in *ARR5* expression level in the shoot after tZ treatment of the root, indicating root-to-shoot cytokinin transport. These findings indicate that *ABCC4* is important for local cytokinin transport within the root tip, while it does not appear to influence long-distance transport to the shoots.

Taken together, these experiments uncover the central role of ABCC4 in cytokinin distribution in the root tips, potentially helping to set the position of the proximal root apical meristem end, also known as the transition domain (Fig. 1). Combined with previous knowledge about other cytokinin transporters, the results presented by Uragami et al. (2024) help to visualize a clearer landscape of cytokinin transport and action across the plant. However, key questions remain, such as how ABCC4-mediated efflux is coupled with other cytokinin transporters or other hormonal pathways, like auxin, to establish the transition domain. Moreover, given *ABCC4*'s broad expression, its role in other developmental processes needs to be addressed. Elucidating the overall role of cytokinins in plants will advance our understanding of plant biology and pave the way for developing new technologies for future applications in plant biotechnology.

## Data Availability

No new data were generated or analysed in support of this article.
